# The induction of tumour cell adhesiveness and intercellular junctions by a glycoprotein of rat ascites hepatoma cell surface.

**DOI:** 10.1038/bjc.1976.188

**Published:** 1976-10

**Authors:** Y. Ishimaru, K. Kudo, H. Ishihara, H. Hayashi

## Abstract

**Images:**


Br. J. Cancer (1976) 34, 426

### THE INDUCTION OF TUMOUR CELL ADHESIVENESS AND INTERCELLULAR JUNCTIONS BY A GLYCOPROTEIN OF RAT ASCITES HEPATOMA CELL SURFACE*

Y. ISHIMARU, K. KUDO, H. ISHIHARA AND H. HAYASHI

*From the Department of Pathology, Kumamoto University Medical School, Kumamoto* 860, *Japan*

Received 5 May 1976 Accepted 22 June 1976

**Summary.—Rat ascites hepatoma AH109A cells (present as a free form *in vivo*) can aggregate and then develop well-defined tripartite junctional complexes, including intermediate junctions, desmosomes and focal tight junctions, on incubation with a glycoprotein separated from rat ascites hepatoma AH136B cells (forming cell islands *in vivo*). The development of binding structures was strongly inhibited by actinomycin D. AH109A cells or rat ascites hepatoma YS cells (present as a free form *in vivo*) previously treated with the glycoprotein for 24 h, when inoculated i.p., proliferated as free cells in the ascitic fluid, like the untreated cells. AH109A cells actively proliferating in the skin do not form any junctional complexes. The reason for the failure of island formation by AH109A cells or YS cells *in vivo* is discussed.**

As PREVIOUSLY DESCRIBED (Kudo *et al.*, 1974), a thermostable glycoprotein, capable of inducing tumour cell aggregation, has been separated from rat ascites hepatoma AH136B cells forming cell islands *in vivo*, and partially purified by chromatography. This aggregation-promoting factor (APF) was non-cytotoxic and clearly effective for aggregation of dissociated AH136B cells as well as SV40 transformed cells, but not for normal rat liver cells and red blood cells. It has further been shown that the APF can cause both aggregation of rat ascites hepatoma AH109A cells present in a free form *in vivo* and adhesiveness of the cells, characterized by gradual development of known binding structures, including intermediate junctions, desmosomes and focal tight junctions (Ishimaru, Ishihara and Hayashi, 1975). On the basis of preliminary observations indicating that the amount of APF separated from AH109A cells is apparently smaller than that of APF from AH136B cells (Kudo, Hanaoka and Hayashi, 1972), it was of interest to investigate the reason why most of AH109A cells are present in a free form *in vivo*. The purpose of the present communication is to describe the electron microscopic evidence that AH109A cells only develop the binding structures when the APF is applied to the cells in a sufficient amount.

### MATERIALS AND METHODS

*Rat ascites hepatoma.*—Rat ascites hepatomas AH136B, AH109A and YS have been maintained in our laboratory by routine passage of $1 \times 10^6$ AH136B cells, $2 \times 10^6$ AH109A cells or $2 \times 10^6$ YS cells injected i.p. into 80–100-g male rats of Donryu strain. Most (about 98%) of the AH136B cells were found to form cell islands of varying size *in vivo*. On the other hand, most (about 95%) of the AH109A cells and all of the YS cells were revealed to be free *in vivo*.

*Isolation of aggregation-promoting factor (APF).*—This was performed essentially by the method previously described by Kudo *et al.* (1974). APF was released from $15 \times 10^8$

* No. 5 of a series on Tumour Cell Aggregation-Promoting Factor

Correspondence: Professor H. Hayashi, Department of Pathology, Kumamoto University Medical School, Kumamoto 860, Japan.

AH136B cells, suspended in Hanks' balanced salt solution (free of calcium and magnesium) in the cold, by treatment with 50 gentle "pipettings" and partially purified by chromatography using DEAE-Sephadex and gel filtration using Bio-gel. The substance was then made up in Earle's MEM at desired concentrations, measured as absorbancy at 280 nm/ml. Before use, APF solution was filtered through Millipore filters (pore size 0·3 μm).

*Preparation of cell suspension.*—The cell suspension was prepared as follows (Ishimaru *et al.*, 1975): ascitic fluid (20 ml) was withdrawn by i.p. puncture 7 days after inoculation of AH109A cells or 5 days after inoculation of YS cells and diluted 1 : 5 with 0·45% NaCl solution. The cell suspension was kept at room temperature for 60 min to allow red blood cells to separate, and tumour cells were sedimented by centrifugation at 25 *g* for 10 min. After washing with 0·45% NaCl, the cells were finally suspended at a concentration of $2 \times 10^6$ cells/ml in Earle's MEM containing 20% normal rat serum. APF caused aggregation of either AH109A cells or YS cells, when tested at a concentration of $2 \times 10^6$ cells/ml, similar to that observed with AH136B cells at a concentration of $2 \times 10^5$ cells/ml (Kudo *et al.*, 1974).

In vitro *induction of tumour cell aggregation.*—This was carried out essentially by the method previously described (Kudo *et al.*, 1974). Equal volumes (1·5 ml) of APF solution (0·15 mg/ml) and tumour cell suspension were mixed in a Falcon tube (1·5 × 9·5 cm) and incubated at 37°C in a roller tube culture apparatus, model Te-Her (Hirasawa Co., Tokyo, Japan) run at one rotation/8 min. At intervals of 12 and 24 h after addition of APF, cell aggregates formed were removed by a pipette from the Falcon tubes for light and electron microscopic examination.

Experimental cultures were assayed as follows: (*a*) 2 h after incubation of tumour cells with APF at 37°C, the cell aggregates formed were gently washed 5 times with Earle's MEM and then re-incubated in fresh medium (Earle's MEM containing 20% normal rat serum) free of APF for a further 22 h and (*b*) tumour cells were incubated in APF solution containing 0·75 μg of actinomycin D (P–L Biochemicals, Inc., Milwaukee, Wis., U.S.A.) at 37°C for 24 h. Actinomycin D at the concentration (0·5 μg/ml) tested induced no morphological change visible by light or electron microscope over a 24-h period.

*Subcutaneous transplantation of AH109A cells.*—$1{\cdot}5 \times 10^7$ of AH109A cells were transplanted s.c. into the backs of normal Donryu rats (Hayashi *et al.*, 1970; Yoshida *et al.*, 1970). Seven days later, growing tumours were removed for light and electron microscopic observation.

*Electron microscopy.*—Immediately after removal from Falcon tubes, the aggregated cells were placed in cold 4% glutaraldehyde in 0·1M S-collidine buffer (pH 7·3–7·4) for 45 min. The cells were rinsed with cold 0·1 M S-collidine buffer and then fixed in cold 2% osmium tetroxide in 0·1M S-collidine buffer for 45 min. Tumour tissues were similarly treated but for 90 min in both steps. The fixed material was stained with 2% uranyl-acetate in distilled water for 60 min at room temperature to enhance membrane and fibrillar structures. The samples were dehydrated with graded alcohol and embedded in Epon 812 in the usual way. Thin sections cut with a Porter-Blum MT-1 microtome (Ivan Sorvall Inc., Norwalk, Conn., U.S.A.) were stained with lead acetate, mounted on 150-mesh grids coated with collodion film and examined in a Hitachi-12 A electron microscope (Hitachi Ltd, Tokyo, Japan). Measurements were made with a magnifying measuring eyepiece on print of known enlargement. Thick sections were also prepared for light microscopy and stained with toluidine blue.

### RESULTS

### I. *Light microscopic observation of tumour cell aggregation*

Equal volumes (1·5 ml) of APF solution (0·15 mg/ml) and tumour cell suspension were mixed and incubated at 37°C. Induction of macroscopic aggregation ofA H109A cells or YS cells at a concentration of $2 \times 10^6$ cells/ml became visible after 10 min of incubation. On further incubation the cell aggregates became larger and fused together. After 30 min incubation the aggregates settled to the bottom of the Falcon tubes, as previously described (Ishimaru *et al.*, 1975). The cell aggregates were removed after 12 and 24 h of contact with APF,

FIG. 1*a*.—Light microphotograph of aggregated AH109A cells after 24 h incubation with APF. Stained with aceto-gentian violet. Similar results were obtained with YS cells. × 45.

FIG. 1*b*.—Light microphotograph of aggregated AH109A cells after 2 h incubation with APF and a further 22 h incubation without APF. × 45.

FIG. 1*c*.—Light microphotograph of aggregated AH109A cells in 24 h incubation with APF and actinomycin D. The cell aggregates show a somewhat irregular shape, possibly due to loose arrangement of the cells. × 45.

fixed and stained with aceto-gentian violet solution prepared by the method of Yoshida *et al.* (1955). Microscopically, the aggregated cells showed a tendency to arrange themselves in a concentric pattern (Fig. 1*a*). No cell aggregation occurred in the absence of APF. No difference in the induction of aggregation between AH109A cells and YS cells was observed. On the basis of these observations, the following experiments were preformed.

(*a*) *AH109A cell aggregation in 2 h contact with APF*.—Equal volumes (1·5 ml) of APF solution (0·15 mg/ml) and AH109A cell suspension were mixed and incubated at 37°C. After 2 h the cell aggregates formed were washed and then re-incubated in the medium free of APF for 22 h. Microscopically, the cell aggregates in 22 h of re-incubation were almost indistinguishable in their shapes or in the arrangement of individual cells from those observed in the control cultures (Fig. 1*b*).

(*b*) *Effect of actinomycin D on AH109A cell aggregation*.—Equal volumes (1·5 ml) of tumour cell suspension and APF solution containing 0·75 $\mu$g of actinomycin D were mixed and incubated at 37°C for 24 h. The cell aggregates formed resembled those seen in the control cultures. However, the microscopic cell aggregates seemed to be somewhat different from those observed in the control cultures; their shapes were irregular, and individual cells were loosely arranged in the cell aggregates (Fig. 1*c*).

*II. Electron microscopic observation of AH109A cell adhesiveness*

On the basis of observations previously described (Ishimaru *et al.*, 1975), electron microscopic features of AH109A cell adhesiveness at 12 and 24 h after contact with APF were re-examined and reconfirmed. The frequencies of simple apposition, intermediate junction, desmosome and focal tight junction observed at 24 h after contact with APF were in the ratio of 10 : 6 : 4 : 0·2 when counted for 50 cells. These experiments provided the control electron microscopic pictures for the development of binding structures in the experimental cultures.

FIG. 2*a*.—Adherent AH109A cells observed after 2 h cultivation with APF and 22 h without. The adhesiveness of these cells is clearly close and characteristic. Surface regions showing close contact are increased. S →, simple apposition. I →, intermediate junction. D →, desmosome. F →, focal tight junction. × 4875.

FIG. 2*b*.—Desmosomes (indicated by arrow) observed in adherent AH109A cells after 2 h cultivation with APF and 22 h without. × 19,000.

(*a*) *Cell adhesiveness after 2-h contact with APF*.—After 2 h of contact with APF (0·15 mg/ml), the cell aggregates formed were washed and re-incubated in medium free of APF for 22 h. After 22 h of re-incubation, cell adhesiveness became more pronounced as compared with control cultures after a 12 h contact with APF, with a clearly observable increase in the cell surface regions showing close contact

FIG. 3.—Intermediate junction (I, indicated by arrow) and focal tight junction (F, indicated by arrow) observed in adherent AH109A cells after 2 h cultivation with APF and 22 h without. I consists of 2 outer leaflets disposed in a parallel fashion and separated by a space of 10–15 nm with low electron density. In the cytoplasm subadjacent to the inner leaflet, electron-dense materials are seen. F is characterized by punctate fusion of outer leaflets. × 78,000.

(Fig. 2*a*, *b*). In addition to simple apposition, as described below, the cell contact was characterized by an increase of intermediate junctions, desmosomes and focal tight junctions. The intermediate junctions consisted of 2 outer leaflets disposed in a parallel fashion and separated by intercellular space of less than 20 nm occupied by homogeneous, apparently amorphous materials of low density, which resemble those described by Farquhar and Palade (1963) (Fig. 3). The cytoplasm subadjacent to the inner leaflets showed moderate electron density.

The desmosome-like structures observed at this stage consisted of 2 outer leaflets running in a parallel fashion and separated by an intercellular space of about 17 nm containing a central disc of electron-dense materials. In the cytoplasm subadjacent to each inner leaflet, electron-dense laminar plaques running parallel to the inner leaflets were observed (Farquhar and Palade, 1963; Trelstad, Hay and Revel, 1967; Lentz and Trinkaus, 1971). Such structures seemed to be divided into 3 types: (1) desmosomes were characterized by 2 electron-dense laminar plaques which were not accompanied by endoplasmic fibrils, like those observed after 12 h in contact with APF in control cultures (Ishimaru *et al.*, 1975); (2) desmosomes characterized by one distinct laminar plaque and one obscure laminar plaque accompanied by a few endoplasmic fibrils (Fig. 4*a*); and (3) well defined desmosomes characterized by one distinct laminar plaque accompanied by prominant endoplasmic fibrils (Fig. 4*b*). In general, the outer leaflets seemed to have higher electron density than the inner leaflets. Well-defined focal tight junctions, as described by Trelstad *et al.* (1967), were occasionally observed in the limited surface regions of close cell contact (Fig. 3). The frequency of simple apposition, intermediate junction, desmosome and focal tight junction observed at this stage seemed to be in the ratio of 10 : 6 : 4 : 0·15 respectively when counted for 50 cells. These electron microscopic pictures of cell adhesiveness were essentially indistinguishable from those revealed at 24 h after contact with APF in the control cultures.

(*b*) *Effect of actinomycin D on cell adhesiveness by APF*.—When actinomycin D (0·75 μg) was added with APF (0·15 mg/ml) during 24 h of cultivation, development of the binding structures noted above

FIG. 4*a*.—Desmosome observed in adherent AH109A cells after 2 h cultivation with APF and 22 h without. It is characterized by 2 electron-dense laminar plaques ($P_1$ and $P_2$); $P_1$ is distinct, but $P_2$ is less distinct. Two outer leaflets are separated by a space of about 17 nm showing central disc of electron-dense materials. Only a few endoplasmic fibrils are seen. × 60,000.

FIG. 4*b*.—Desmosome observed in adherent AH109A cells after 2 h cultivation with APF and 22 h without, which is characterized by one distinct laminar plaque (P). Prominent endoplasmic fibrils (indicated by arrow) are related to the plaque. × 60,000.

was strongly inhibited. In general, intercellular spaces became larger and areas of cellular apposition became smaller; the cell surface regions showing close contact were apparently decreased as compared with those seen in the control cultures (Fig. 5). The binding structures observed in areas of close cell contact were mostly simple apposition of plasma membranes as described by Farquhar and Palade (1963). Apposed plasma membranes were separated by a space of 10–30 nm with no electron density (Fig. 6). The structure consisted of 2 outer leaflets disposed in a parallel fashion, showing focal membrane undulation of varying degrees. At this stage of cell contact, intermediate junction was rarely found, and no structures resembling desmosomes or focal tight junctions were observable.

Fig. 5.—AH109A cells observed after 24 h incubation with APF and actinomycin D. Intercellular spaces are larger, and areas of cellular apposition are smaller. The cell surface regions showing close contact are apparently decreased. The areas of close contact consist of only simple apposition (indicated by arrow). No intermediate junction, desmosome and focal tight junction. × 4650.

Fig. 6.—Simple apposition observed in AH109A cells in 24 h incubation with APF and actinomycin D. Two plasma membranes are separated by intercellular space of 20–30 nm. The specialized junctional structures are not seen. × 40,000.

FIG. 7a.—AH109A cells proliferated for 7 days after s.c. transplantation. The surface regions of the cells show close contact consisting of only simple apposition (indicated by arrow). × 8000.

*III. Intraperitoneal inoculation of AH109A and YS cells previously treated with APF*

Equal volumes (1·5 ml) of tumour cell suspension (containing $2 \times 10^6$ AH109A cells/ml or $2 \times 10^6$ YS cells/ml) and APF solution (0·5 mg/ml) were mixed and incubated for 24 h. The aggregated cells were carefully collected by a pipette (3 mm in diameter) and inoculated i.p. through a small surgical opening (1·0 cm long) in the abdominal skin of 3 male Donryu rats (80–100 g) of each experimental group. The incision was closed immediately after inoculation. In control experiments, the APF solution was replaced by the Hanks' balanced salt solution.

Of the animals given i.p. AH109A cells previously treated with APF for 24 h, 2 died on the 14th day and 1 on the 15th day after inoculation. Of the animals which were inoculated with AH109A cells without APF, 2 died on the 13th day and 1 on the 12th day. APF treatment thus had no significant effect upon the survival time of the animals. Just before or immediately after death, ascitic fluid was withdrawn from each animal and the cells examined microscopically. Most (about 94%) of the cells in the ascitic fluid were found to be free; no morphological differences were observed between APF-treated AH109A cells and controls.

The animals which were given i.p. YS cells treated with APF for 24 h died as follows: 2 on the 8th day and 1 on the 9th day. Of the control animals inoculated with YS cells untreated with APF, 1 died on the 6th day, 2 on the 7th day. No morphological differences were observed between APF-treated and control YS cells

FIG. 7b.—Simple apposition observed in actively proliferating AH109A cells in the skin. Two plasma membranes are disposed in parallel and separated by intercellular space of about 20 nm. × 45,000.

FIG. 7c.—A few collagen fibres (indicated by arrow) in the wide intercellular space of adjacent AH109A cells in the skin. × 12,000.

in the ascitic fluid and all the cells were found to be free.

*IV. Electron microscopic observation of AH109A cells grown in skin*

The growing tumour tissues were removed 7 days after s.c. transplantation of AH109A cells for electron microscopic examination. Pronounced proliferation of the cells was observed (Yoshida *et al.*, 1970). The cell surface regions of actively proliferating AH109A cells clearly showed close contact, but the cell contact was only simple apposition (Fig. 7*a*, *b*). No intermediate junction, desmosome and focal tight junction were found. A few collagen fibres were occasionally revealed between AH109A cells when the cells showed a wide intercellular space (Fig. 7*c*).

DISCUSSION

The results presented here demonstrate that APF induces not only a strong cell aggregation but also a distinct cell adhesiveness, characterized by development of well-defined binding structures in the adherent cells, when AH109A cells in a free form were treated with APF for only 2 h. The electron microscopic features of the adherent AH109A cells were essentially indistinguishable from those observed in the adherent AH109A cells in a long (24 h) contact with APF; the frequency of simple apposition, intermediate junction, desmosome and focal tight junction being in the ratio of 10 : 6 : 4 : 0·15. This strongly suggests that APF causes aggregation of tumour cells, with the development of binding structures in the aggregated cells. Thus APF may be involved in the development of such binding structures leading to tumour cell adhesiveness. Further observations demonstrate that APF, in the presence of actinomycin D (0·75 $\mu$g) for 24 h of incubation, induces aggregation of AH109A cells, but without the binding structures observed in the absence of actinomycin D. It seems reasonable that the failure to develop binding structures may be associated with the inhibition of RNA synthesis by actinomycin D (Reich *et al.*, 1961; Anservin, 1965).

The development of binding structures has been analysed in detailed electron microscopic studies on re-aggregation of trypsinized chick embryonal cells; various re-aggregated cells (including pigmented retinal, cardiac, hepatic and corneal cells) developed intermediate junction and desmosome in 19 or 24 h of cultivation (Armstrong, 1970; Sheffield and Moscona, 1970; Overton, 1974) and re-aggregated cells from the optic lobe developed intermediate junction and focal tight junction after 24-h cultivation (Adler, 1971). It was of interest to note that the duration necessary for development of binding structures in these embryonal cells was comparable to that necessary for development of tripartite junctional complexes in the AH109A cells after contact with APF (Ishimaru *et al.*, 1975). Since the dissociated cells were able to re-aggregate without addition of any cell-aggregating substance, it was considered that these cells themselves may synthesize some cell-aggregating substance in culture (Hausman and Moscona, 1973). Recently, it has been shown in culture that dissociated liver cells from normal adult rats can re-aggregate and re-establish many of the intercellular junctions typical of adult liver *in vivo* (Alwen and Lawn, 1974). On the basis of morphological observations that the alignment of cysternae of endoplasmic reticulum beneath planar surfaces of contact between hepatocytes occurred in the early stages, they postulated that materials associated with cell adhesion were secreted during the process.

Re-aggregation of dissociated embryonal cells described above has been shown to be inhibited by actinomycin D, puromycin, or proflavine. Thus, agents which inhibit RNA or protein synthesis block the synthesis of cell-aggregating substance in the embryonal cells (Moscona and Moscona, 1963; Hausman and Moscona, 1973). Island formation of AH136B cells *in vivo* involves the formation of clearly characterized intercellular junctions (Ishihara, Ishimaru and Hayashi, 1976). Accordingly, it is suggested that AH136B cells themselves may produce the APF required for the development of binding structures, resulting in the formation of cell islands. The APF used in the present experiment was separated from AH 136B cells.

In contrast to AH136B cells, AH109A cells were found to be free in the ascitic fluid *in vivo*; and even in the close contact regions of actively proliferating AH109A cells in tissue, no development of intermediate junction, desmosome and focal tight junction was observed. As mentioned above, the AH109A cells, however, aggregated in the presence of APF from AH136B cells and then developed well-defined binding structures. This strongly suggests that the AH109A cells have the ability to synthesize proteins necessary for development of binding structures, provided APF is present. Accordingly, it is suggested that the AH109A cells lack the ability to produce APF in an amount sufficient for the development of binding structures. This is also supported by the evidence that AH109A cells or YS cells previously treated with APF for 24 h, when inoculated i.p., proliferate as free cells in the ascitic fluid, like untreated AH109A cells or YS cells. As previously suggested (Kudo, Hanaoka and Hayashi, 1972), the amount of APF which was separated from AH109A cells by the previous method (Kudo *et al.*, 1974), was apparently smaller than that of APF separated from AH136B cells. Since the APF has been highly purified by immunoadsorbent chromatography (Kudo, Hanaoka and Hayashi, 1976*a*, *b*), a quantitative estimation by radioimmunoassay of APF from various types of rat ascites hepatoma cells is desirable.

We would like to record our appreciation to Dr Y. Hanaoka for the isolation of an aggregation-promoting factor from rat ascites hepatoma cells. This work was supported in part by a special grant for cancer research from the Japanese Ministry of Education and by a grant from the Shionogi Biological Institute, Osaka, Japan.

### REFERENCES

ADLER, R. (1971) Ultrastructural Changes Associated with Invagination Phenomenon in Embryonic Neural Aggregates. *Expl Cell Res.*, **68**, 395.

ALWEN, J. & LAWN, A. M. (1974) The Reaggregation of Adult Rat Liver Cells Maintained *in vitro*. *Expl Cell Res.*, **89**, 197.

ANSERVIN, K. D. (1965) The Effect of RNA Synthesis Inhibition and of Protein Synthesis Inhibition on Embryonic Induction and Cell Differentiation in *Rana pipiens*. *J. Morphol.*, **117**, 171.

ARMSTRONG, P. B. (1970) A Fine Structural Study of Adhesive Cell Junctions in Heterotypic Cell Aggregates. *J. Cell Biol.*, **47**, 197.

FARQUHAR, M. G. & PALADE, G. E. (1963) Junctional Complexes in Various Epithelia. *J. Cell Biol.*, **17**, 375.

HAUSMAN, R. E. & MOSCONA, A. A. (1973) Cell-Surface Interactions: Differential Inhibition by Proflavine of Embryonic Cell Aggregation and Production of Specific Cell-Aggregating Factor. *Proc. natn. Acad. Sci., U.S.A.*, **70**, 3111.

HAYASHI, H., YOSHIDA, K., OZAKI, T. & USHIJIMA, K. (1970) Chemotactic Factor Associated with Invasion of Cancer Cells. *Nature, Lond.*, **226**, 1174.

ISHIHARA, H., ISHIMARU, Y. & HAYASHI, H. (1976) An Electron Microscopic Study of Binding Structures in Rat Ascites Hepatoma Cells. *Trans. Soc. Path. Jap.*, **65** (in Japanese). (Abstract).

ISHIMARU, Y., ISHIHARA, H. & HAYASHI, H. (1975) An Electron Microscopic Study of Tumour Cell Adhesiveness Induced by Aggregation Promoting Factor from Rat Ascites Hepatoma Cells. *Br. J. Cancer*, **31**, 207.

KUDO, K., HANAOKA, Y. & HAYASHI, H. (1972) Aggregation Factor from Ascites Fluid and Serum of Tumour-bearing Rats and its Origin. *Proc. Jap. Cancer Assoc.*, **29**, 80 (in Japanese). (Abstract).

KUDO, K., HANAOKA, Y. & HAYASHI, H. (1976*a*) Characterization of Tumour Cell Aggregation Promoting Factor from Rat Ascites Hepatoma Cells: Separation of Two Factors with Different Antigenic Property. *Br. J. Cancer*, **33**, 79.

KUDO, K., HANAOKA, Y. & HAYASHI, H. (1976*b*) Purification of a Tumour Cell Aggregation Promoting Factor Associated with Rat Ascites Hepatoma Cell Surface. *Br. J. Cancer*, **34**, 88.

KUDO, K., TASAKI, I., HANAOKA, Y. & HAYASHI, H. (1974) A Tumour Cell Aggregation Promoting Substance from Rat Ascites Hepatoma Cells. *Br. J. Cancer*, **30**, 549.

LENTZ, T. L. & TRINKAUS, J. P. (1971) Differentiation of the Junctional Complex of Surface Cells in the Developing *Fundulus* Blastoderm. *J. Cell Biol.*, **48**, 455.

MOSCONA, M. H. & MOSCONA, A. A. (1963) Inhibition of Adhesiveness and Aggregation of Dissociated Cells by Inhibitors of Protein and RNA Synthesis. *Science, N.Y.*, **142**, 1070.

OVERTON, J. (1974) Selective Formation of Desmosomes in Chick Cell Reaggregates. *Develop. Biol.*, **39**, 210.

REICH, E., FRANKLIN, R. M., SHATKIN, A. J. & TATUM, E. L. (1961) Effect of Actinomycin D on Cellular Nucleic Acid Synthesis and Virus Production. *Science, N.Y.*, **134**, 556.

SHEFFIELD, J. B. & MOSCONA, A. A. (1970) Electron Microscopic Analysis of Aggregation of Embryonic Cells: The Structures and Differentiation of Aggregates of Neural Retina Cells. *Develop. Biol.*, **23**, 36.

TRELSTAD, R. L., HAY, E. D. & REVEL, J. P. (1967) Cell Contact During Early Morphogenesis in the Chick Embryo. *Develop. Biol.*, **16**, 78.

YOSHIDA, T., ISAKA, H., NAKAMURA, H., ODASHIMA, S. & SATOH, H. (1955) Studies on Rat Ascites Hepatoma Cells. *Trans. Soc. Path. Jap.*, **44**, 407 (in Japanese). (Abstract).

YOSHIDA, H., OZAKI, T., USHIJIMA, K. & HAYASHI, H. (1970) Studies on the Mechanisms of Invasion in Cancer. I. Isolation and Purification of a Factor Chemotactic for Cancer Cells. *Int. J. Cancer*, **6**, 123.


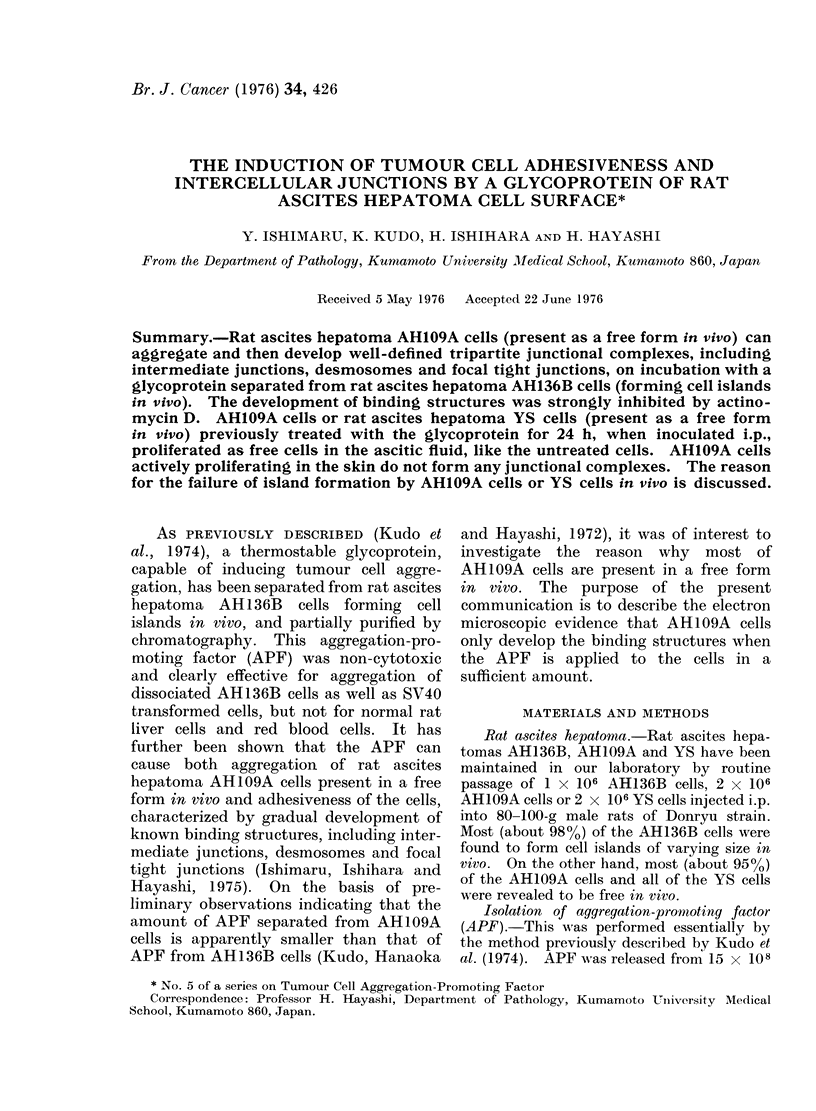

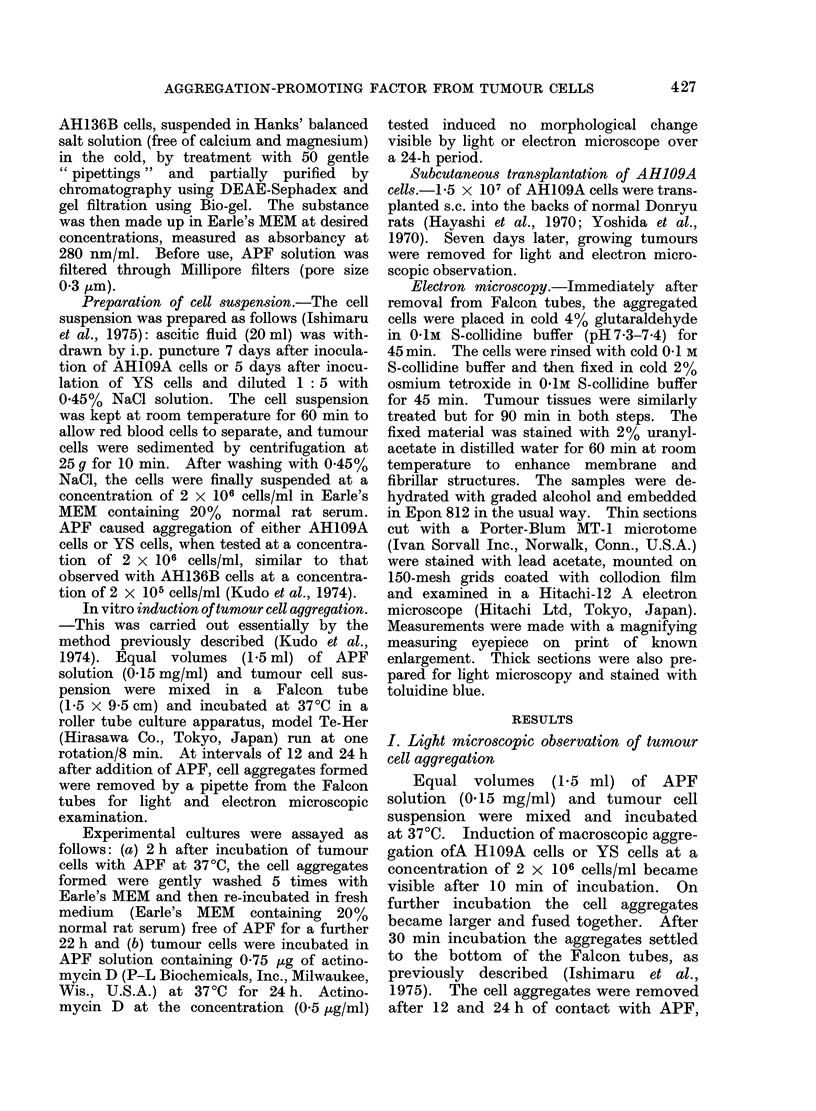

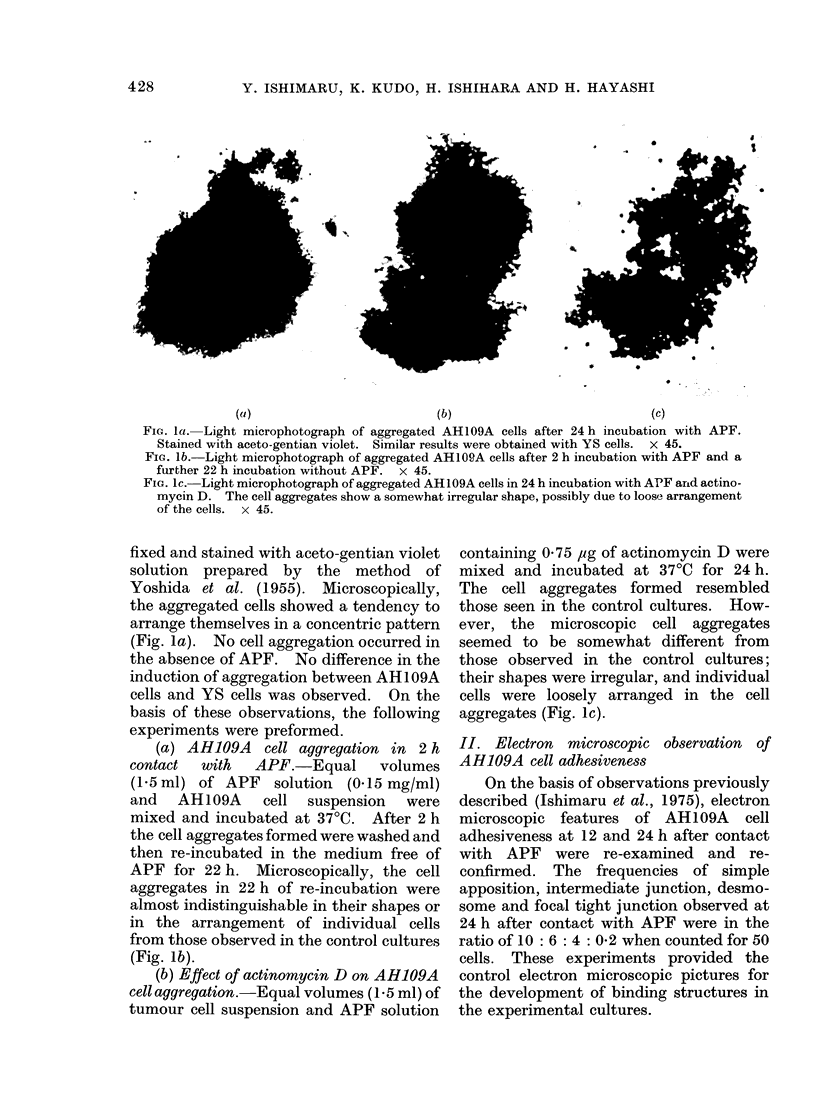

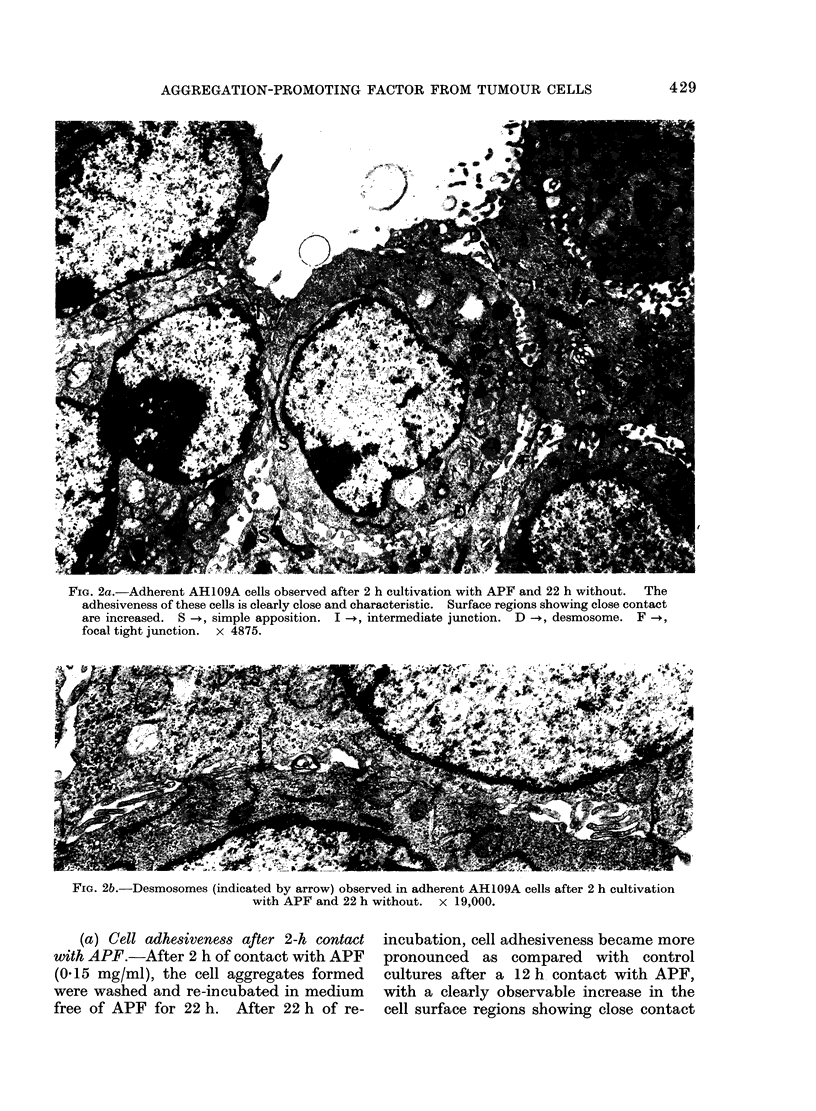

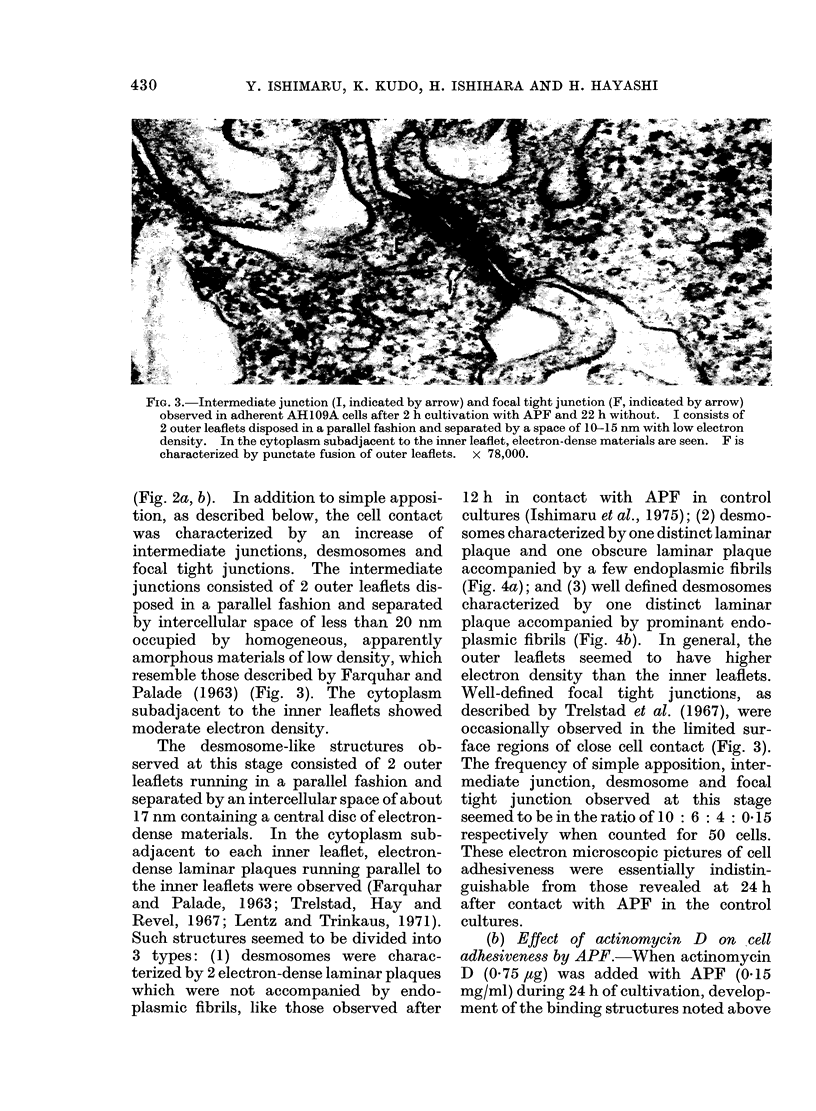

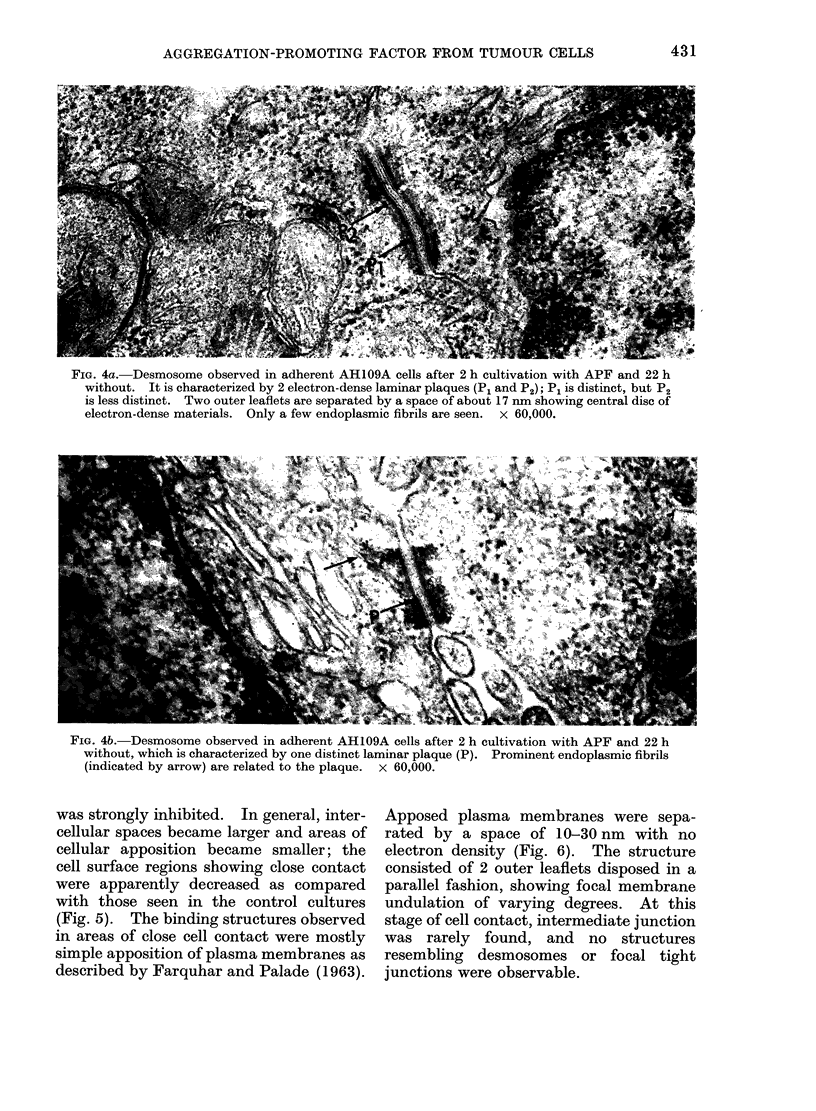

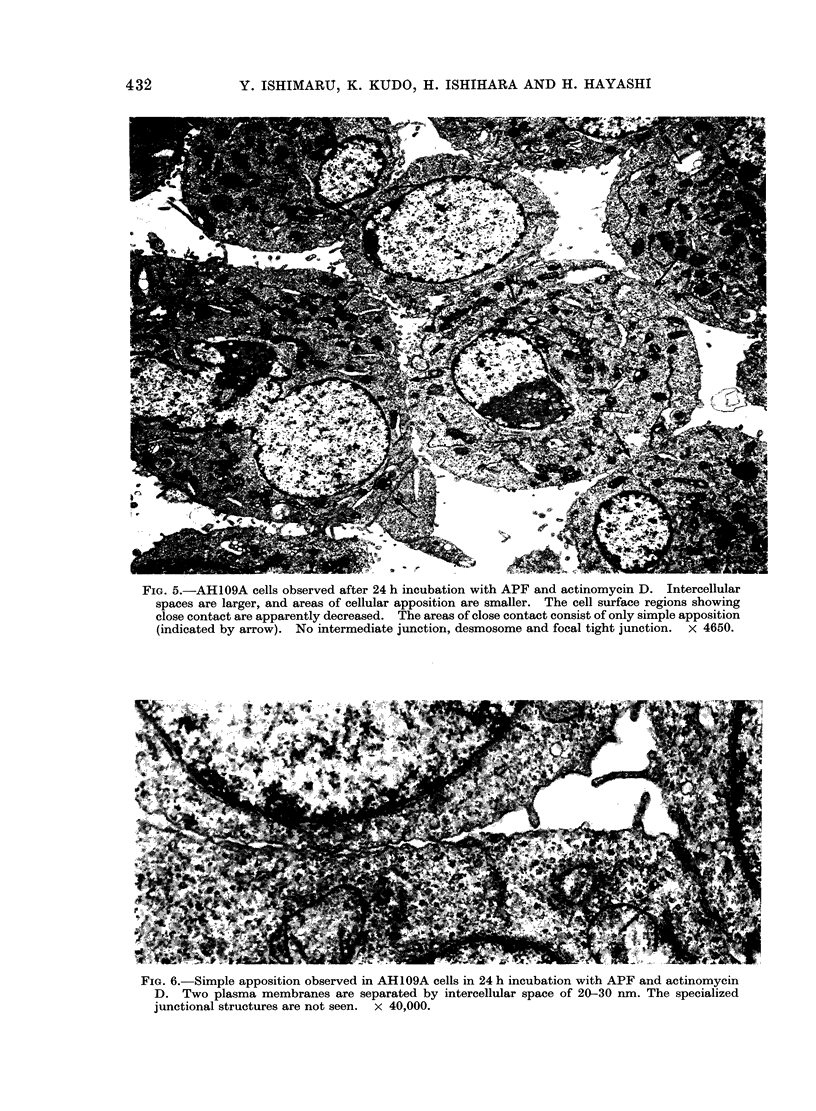

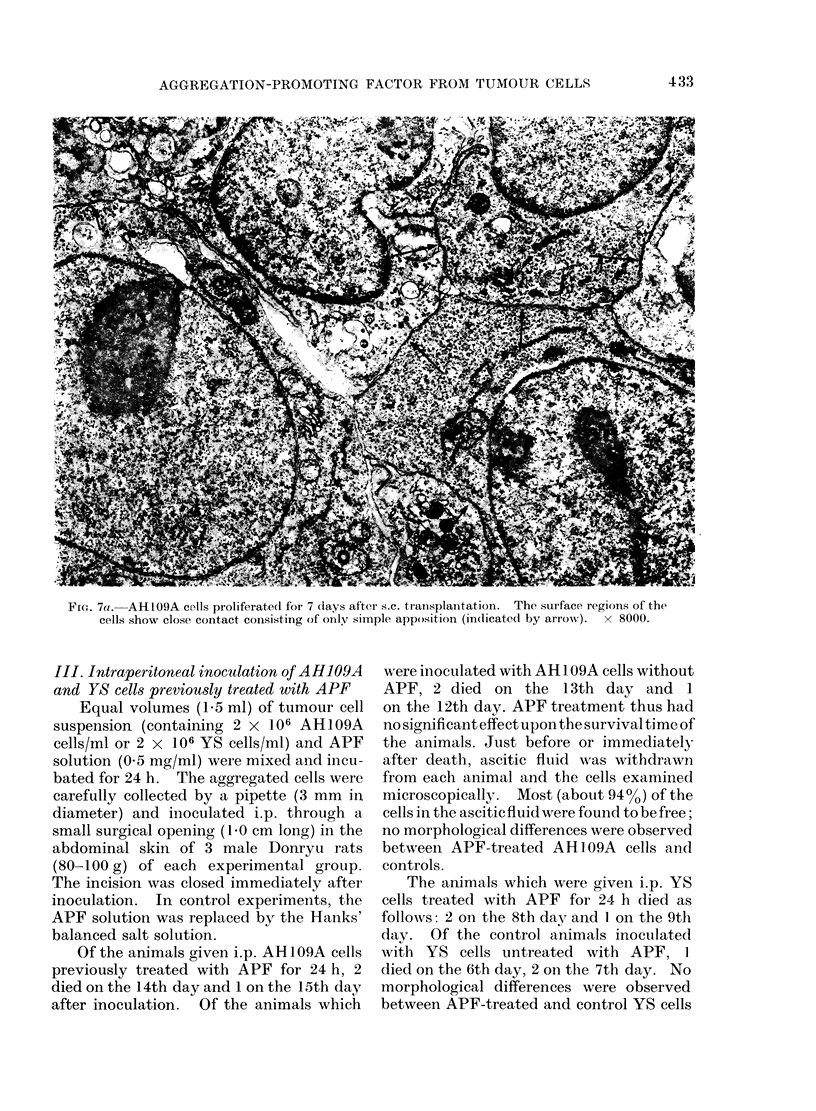

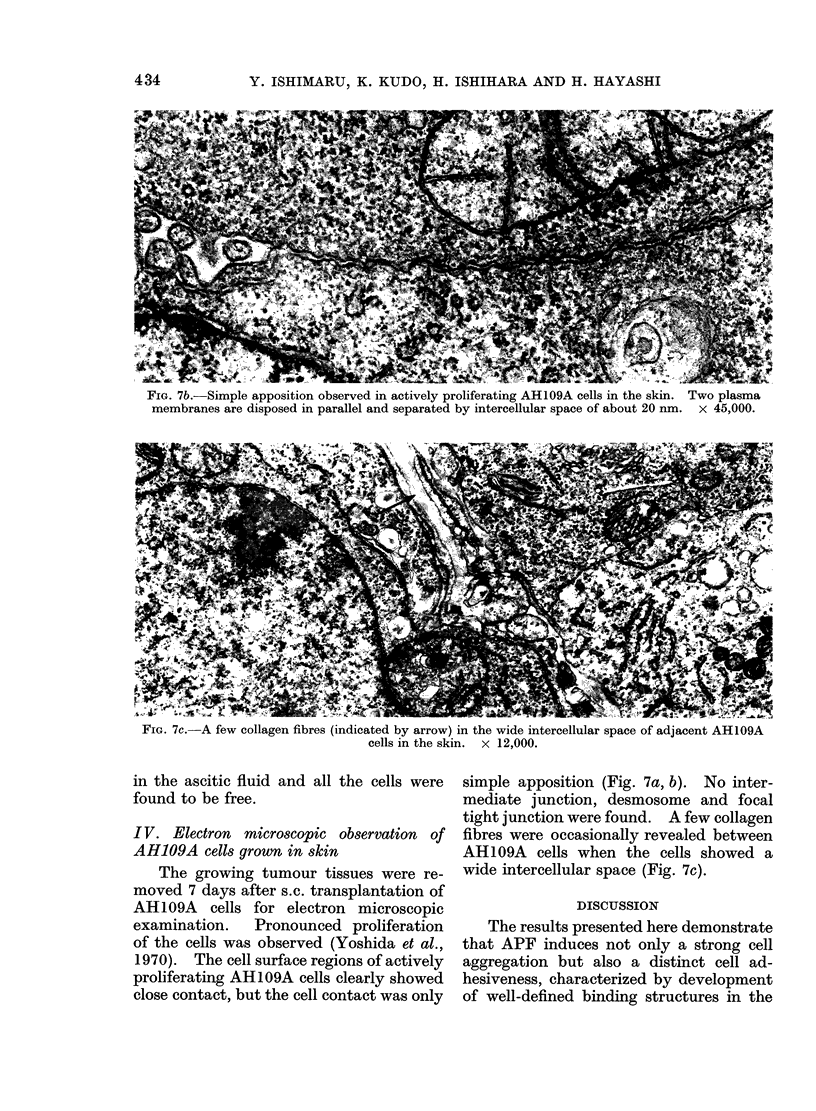

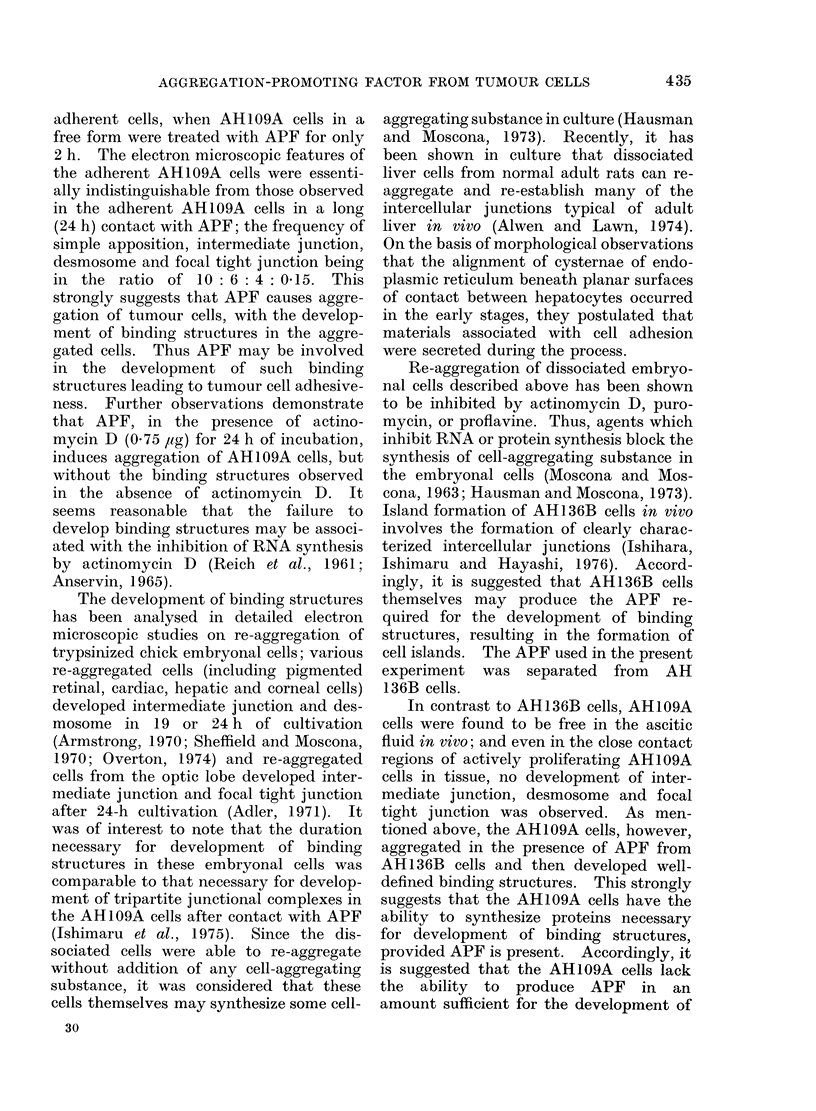

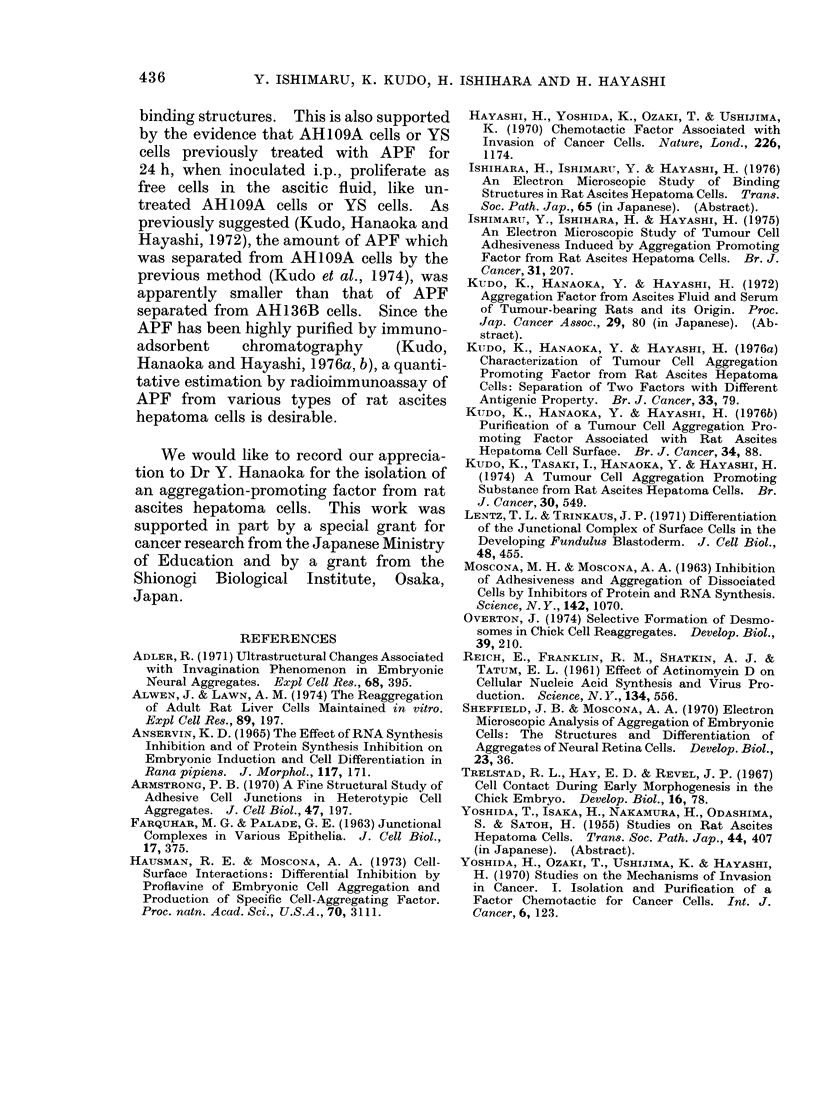


## References

[OCR_00691] Adler R. (1971). Ultrastructural changes associated with an invagination phenomenon in embryonic neural aggregates.. Exp Cell Res.

[OCR_00696] Alwen J., Lawn A. M. (1974). The reaggregation of adult rat liver cells maintained in vitro.. Exp Cell Res.

[OCR_00701] Ansevin K. D. (1965). The effects of RNA synthesis inhibition and of protein synthesis inhibition on embryonic induction and cell differentiation in Rana pipiens.. J Morphol.

[OCR_00707] Armstrong P. B. (1970). A fine structural study of adhesive cell junctions in heterotypic cell aggregates.. J Cell Biol.

[OCR_00712] FARQUHAR M. G., PALADE G. E. (1963). Junctional complexes in various epithelia.. J Cell Biol.

[OCR_00717] Hausman R. E., Moscona A. A. (1973). Cell-surface interactions: differential inhibition by proflavine of embryonic cell aggregation and production of specific cell-aggregating factor.. Proc Natl Acad Sci U S A.

[OCR_00736] Ishimaru Y., Ishihara H., Hayashi H. (1975). An electron microscopic study of tumour cell adhesiveness induced by aggregation promoting factor from rat ascites hepatoma cells.. Br J Cancer.

[OCR_00750] Kudo K., Hanaoka Y., Hayashi H. (1976). Characterization of tumour cell aggregation promoting factor from rat ascites hepatoma cells: Separation of two factors with different antigenic property.. Br J Cancer.

[OCR_00757] Kudo K., Hanaoka Y., Hayashi H. (1976). Purification of a tumour cell aggregation-promotin factor associated with rat ascites hepatoma cell surface.. Br J Cancer.

[OCR_00763] Kudo K., Tasaki I., Hanaoka Y., Hayashi H. (1974). A tumour cell aggregation promoting substance from rat ascites hepatoma cells.. Br J Cancer.

[OCR_00769] Lentz T. L., Trinkaus J. P. (1971). Differentiation of the junctional complex of surface cells in the developing Fundulus blastoderm.. J Cell Biol.

[OCR_00775] MOSCONA M. H., MOSCONA A. A. (1963). INHIBITION OF ADHESIVENESS AND AGGREGATION OF DISSOCIATED CELLS BY INHIBITORS OF PROTEIN AND RNA SYNTHESIS.. Science.

[OCR_00781] Overton J. (1974). Selective formation of desmosomes in chick cell reaggregates.. Dev Biol.

[OCR_00786] REICH E., FRANKLIN R. M., SHATKIN A. J., TATUM E. L. (1961). Effect of actinomycin D on cellular nucleic acid synthesis and virus production.. Science.

[OCR_00792] Sheffield J. B., Moscona A. A. (1970). Electron microscopic analysis of aggregation of embryonic cells: the structure and differentiation of aggregates of neural retina cells.. Dev Biol.

[OCR_00799] Trelstad R. L., Hay E. D., Revel J. D. (1967). Cell contact during early morphogenesis in the chick embryo.. Dev Biol.

[OCR_00810] Yoshida K., Ozaki T., Ushijima K., Hayashi H. (1970). Studies on the mechanisms of invasion in cancer. I. Isolation and purification of a factor chemotactic for cancer cells.. Int J Cancer.

